# Diabetes and hypertension are associated with lowered cognitive performance among middle-aged Brazilian adults: cross-sectional analyses nested in the longitudinal Pró-Saúde study

**DOI:** 10.1590/1516-3180.2020.0269.R1.30102020

**Published:** 2021-02-24

**Authors:** Valéria Maria de Azeredo Passos, Carlos Eduardo Raymundo, Flávia Fioruci Bezerra, Eduardo Faerstein

**Affiliations:** I MD, PhD. Associate Professor, Postgraduate Program on Health Science, Faculdade de Ciências Médicas de Minas Gerais (FCMMG), Belo Horizonte (MG), Brazil.; II IT, MSc. Doctoral Student, Instituto de Estudos em Saúde Coletiva (IESC), Universidade Federal do Rio de Janeiro (UFRJ), Rio de Janeiro (RJ), Brazil.; III RD, DSc. Associate Professor, Institute of Nutrition, Universidade do Estado do Rio de Janeiro (UERJ), Rio de Janeiro (RJ), Brazil.; IV MD, PhD. Associate Professor, Institute of Social Medicine, Universidade do Estado do Rio de Janeiro (UERJ), Rio de Janeiro (RJ), Brazil.

**Keywords:** Cognition, Diabetes mellitus, Hypertension, Middle-aged, Brazil, Diabetes, Cardiovascular risk factors, High blood pressures

## Abstract

**BACKGROUND:**

Cardiovascular risk factors are frequently associated with lowered cognitive performance among elderly people, but rarely among middle-aged adults.

**OBJECTIVES:**

To investigate associations between cardiovascular risk factors (age, physical inactivity, smoking, alcohol use, hypertension and diabetes) and lower cognitive performance among middle-aged (45-64 years) Brazilian adults.

**DESIGN AND SETTING:**

Cross-sectional study nested within the Pró-Saúde cohort. From 2,876 baseline study participants (1999), we randomly selected 488 participants and gave them validated and standardized cognitive tests (2012).

**METHODS:**

We used multiple linear and logistic regression analyses to detect associations of cardiovascular risk factors with crude scores in cognitive tests on memory (word test) and executive function (verbal fluency tests), and with overall cognitive performance scores, respectively.

**RESULTS:**

All cognitive test scores presented statistically significant inverse associations with age and direct associations with education. There was no association between lower cognitive performance and smoking or alcohol use. In both 1999 and 2012, after adjusting for sex, age and schooling, being physically active was inversely associated with lower performance regarding late memory. For individuals with diabetes in 1999, there was an association with lower performance regarding executive function, while there was a borderline association for those reporting it only in 2012. Having a diagnosis of hypertension since 1999 was associated with lower performance regarding both memory and executive functions, while reporting hypertension in 2012 was associated with lower performance regarding executive function.

**CONCLUSIONS:**

Aging, low schooling and cardiovascular risk factors may represent life course disadvantages associated with cognitive decline even among middle-aged Brazilian adults.

## INTRODUCTION

Cognitive dysfunction is a major disabling condition among elderly people, which becomes a heavy burden on families and society. From 1990 to 2016 worldwide, the number of individuals with dementia increased from 20.2 million (95% uncertainty interval, UI: 17.4-23.5) to 43.8 million (95% UI: 37.8-51.0), and this 117% increase can mainly be attributed to population growth and aging. Estimates have shown that Brazil had the second highest age-standardized prevalence of dementia (1,037 cases per 100,000 population; 95% UI: 882-1,220) in 2016.^1^

Cardiovascular risk factors are predictors for cognitive decline and dementia among the elderly.^2^ Recently, hypertension and diabetes mellitus were shown to be directly associated with cognitive decline not only among elderly people but also in the middle-aged population in developed countries.^3^^,^^4^^,^^5^ In Brazil, a cross-sectional analysis on around 15,000 Brazilian civil servants aged 35 to 74 years showed that there was a significant association between presence of diabetes and decreased performance both in memory and in executive functions.^6^

Over the last 30 years, although mortality due to cardiovascular diseases has been declining in Brazil, the prevalence of diabetes and hypertension has been rising, along with obesity.^7^

Life expectancy in Brazil has shown a steady increase from 68.4 years in 1990 to 75.2 years in 2016.^8^ These changes are likely to have a profound societal impact in this country, which has marked social inequalities and social and healthcare services that are insufficient to address the problems associated with an aging population.^9^ It is likely that poor social conditions may be responsible for the premature onset and higher burden of dementia in Brazil.^9^

Brazil has high incidence of diabetes, hypertension and dementia. We hypothesized that cardiovascular risk factors, including hypertension and diabetes, might be associated with lowered cognitive performance, not only in the elderly but also among middle-aged adults in Brazil.

## OBJECTIVE

We investigated the hypothesis that cardiovascular risk factors might be associated with lowered cognitive performance among middle-aged adults in Brazil. Our subjects were Brazilian civil servants participating in the Pró-Saúde study, a cohort study in Rio de Janeiro, Brazil, that had the goal of evaluating several dimensions of health-related determinants.^10^

## METHODS

We conducted this cross-sectional study nested within the Pró-Saúde cohort.^10^ From 2,876 participants involved in the baseline studies in 1999, a subsample was randomly selected from all strata: both sexes, two age groups (less than 50 years versus 50 years or over) and two educational levels (less than high school versus high school or more). There was no difference in clinical and sociodemographic characteristics between the participants in this study sample and those of the baseline population (data not shown).

In 2012, cognitive tests were given to 488 participants aged 35-64 years. We analyzed their cognitive performance according to sociodemographic characteristics (sex, age and schooling) and cardiovascular risk factors (smoking, physical inactivity, alcohol use and presence of diabetes and hypertension), which were investigated both in 1999 and in 2012. Since exposures can change over time, the presence of each risk factor was described as present only in 1999 or only in 2012, or present both in 1999 and in 2012. All participants with diabetes in 1999 confirmed that they still had the disease in 2012, and this analysis was therefore presented as cases described just in 2012 or in both 1999 and 2012. The other risk factors presented changes over the period. Thus, these were described as reported just in 1999 or just in 2012, or both in 1999 and in 2012.

We excluded 40 participants (8.8%) who reported having previously had a stroke or who reported use of drugs that can interfere with cognition: antipsychotics, anticonvulsants, antiparkinsonian drugs and anticholinesterase drugs. Cases of previous stroke were excluded based on self-reported morbidity within a medical diagnosis. All participants were told to bring their prescriptions and the packages of any drugs that they had been using during the preceding two weeks.^10^

A standardized questionnaire was applied to investigate whether the participants had smoked at least one hundred cigarettes during their lifetime and how many alcoholic beverages they had consumed over the preceding two weeks. Leisure-time physical activity was assessed via a dichotomous response (yes, no) regarding whether this had been practiced over the previous two weeks.^10^ Hypertension was defined based on reported use of antihypertensive drugs and on systolic blood pressure ≥ 140 mmHg or diastolic blood pressure ≥ 90 mmHg.^11^ Diabetes was defined through reported use of antidiabetic drugs and through fasting glucose level ≥ 126 mg/dl or glycated hemoglobin (HbA1C) level ≥ 6.5%.^12^

Age was classified into a reference category (35-44 years old) and two groups of middle-aged adults (45-54 and 55-64 years old).

We used the international standard classification to stratify education levels: incomplete elementary education (< 8 years); completed elementary education and incomplete secondary education (8-10 years); completed secondary education and incomplete tertiary education (11-14 years); or completed tertiary education (> 14 years).^13^

Validated and standardized cognitive tests to assess cognitive performance were administered in the same order, in a quiet room, by a trained interviewer.^14^ These field researchers applied the cognitive tests after undergoing training consisting of eight hours of theory classes and another eight-hour training period with volunteers, followed by certification.

A word memory test was used to assess the participants’ immediate memory (learning) by asking them to repeat ten unrelated words presented to them three times. Each word was presented for two seconds, in different orders. Memory recall was assessed by asking the participants to say the same ten words after they had completed other tests. Executive function was assessed by means of verbal fluency tests: 1) a semantic test in which the participants were asked to say as many names of animals as possible; and 2) a phonemic test, in which the participants were asked to say words starting with the letter F (phonemic test), for one minute. This battery of cognitive tests has been found to present moderate reliability for memory and good reliability for executive function.^15^^,^^16^

The participants were stratified into two age groups (< 40 and 40+) and four levels of education (< 8, 8-10, 11-14 and > 14 years of schooling), with the aim of enabling adjustments to the scores for associations with age and education. The mean and standard deviation (SD) of the scores from each cognitive test were calculated for each stratum. A composite z-score was calculated by adding the z-scores of the cognitive tests. A composite cognitive test z-score that was one or more SD below the average was considered to represent low cognitive performance.^17^

The statistical analyses were conducted using the STATA software, version 14.0 (Statacorp, College Station, Texas 77845, United States).^18^ Multiple linear regression analysis was used to determine associations between sociodemographic variables and the crude scores of tests. Multiple logistic regression was used to examine associations between cardiovascular risk factors and lowered cognitive performance. Both the multivariate logistic model and the linear regression model were adjusted for sex, age and education.^19^

The Research Ethics Committee of the Institute of Social Medicine, Rio de Janeiro State University, approved the study protocol in October 2011, under the number 0041.0.259.000-11. All participants signed a written informed consent statement.

## RESULTS

Among the participants, women were slightly predominant (51.8%). Most of the participants (56.9%) had had more than 14 years of schooling, and none of them had had less than 8 years of schooling. During the 13 years of follow-up, 18.6% and 2.1% of the participants developed hypertension and diabetes, respectively.

In univariate analysis, women performed better than men regarding learning (immediate memory). Older age, lower schooling level and presence of hypertension were significantly associated with worse performance in all cognitive tests, while diabetes was associated with worse performance in the phonemic verbal fluency test (Table 1).

**Table 1.** Descriptive analysis on cognitive performance according to participants’ characteristics. Pró-Saúde study, Brazil, 2012

Variables	n	%	Learning	Recall	Semantic test	Phonemic test
			Median (interquartile interval)			
Sex						
Male	235	48.2	21 (18-24)	7 (6-8)	21 (16-25)	13 (11-16)
Female	253	51.8	22 (19-24)	7 (6-9)	19 (16-23)	13 (10.8-16)
			P = 0.004^*^	P = 0.049	P = 0.014	P = 0.661
Age group (years)						
35-44	100	20.5	23 (21-26)	8 (7-9)	21 (17-25)	14(11.8-16)
45-54	243	49.8	22 (19-24)	7 (6-8)	21 (16-25)	14 (11-17)
55-64	145	29.7	20 (17-23)	7 (5-8)	18 (14-21)	12 (9-15)
			P < 0.001	P < 0.001	P < 0.001	P < 0.001
Schooling (years)						
8-10	36	7.4	17.5 (16-20)	5 (4-6)	15 (13-20)	10.5 (8-15)
11-14	173	35.7	21 (18-23)	7 (6-8)	19 (15-22)	12 (9-15)
14+	276	56.9	23 (20-25)	8 (6-9)	21 (17-25)	14.0 (12-17)
			P < 0.001	P < 0.001	P < 0.001	P < 0.001
Smoking						
Never	280	62.4	22 (19-24.2)	7.5 (6-9)	20 (16-25)	13 (11-17)
1999	120	26.7	21 (18-23)	7 (6-8)	19.5 (16-23)	13 (10-16)
2012	1	0.2	24 (24-24)	9 (9-9)	25 (25-25)	21 (21-21)
1999 and 2012	48	10.7	20 (17-23)	6 (5-7.2)	18.5 (14.7-22)	12 (9.7-16)
			P = 0.002	P < 0.001	P = 0.048	P = 0.144
Alcohol use						
Never	130	27.7	22 (18-24)	7 (6-8)	19 (16-24)	14 (10.2-17)
1999	55	11.7	22 (19-24)	8 (6-8.8)	21.5 (17-24.8)	13 (10-17)
2012	59	12.5	22 (19-23)	7 (6-9)	19 (16-2.5)	13 (10-15)
1999 and 2012	226	48.1	22 (19-24)	7 (6-8)	20 (16-24)	13 (11-16)
			P = 0.793	P = 0.267	P = 0.312	P = 0.772
Physical activity						
Never	184	39.8	21 (18-24)	7 (5-8)	19 (15-22)	13 (9-15.5)
1999	84	18.3	22 (19-25)	7 (6-8)	21 (16-25)	13 (11-17)
2012	94	20.3	22.5 (19-25)	8 (7-9)	21 (17-24)	14 (11.2-17)
1999 and 2012	100	21.6	22 (19-23)	7 (6-8)	20 (17-25)	13 (11-17)
			P = 0.009	P = 0.002	P = 0.006	P = 0.033
Hypertension						
Never	244	56.1	22 (20-25)	8 (6-9)	20 (16-25)	14 (11-17)
1999	19	4.4	19 (16-23.5)	6 (4-8)	18 (15.5-19)	12 (10.5-13)
2012	81	18.6	21 (19-24)	7 (6-8)	21 (17-24)	13 (11-16)
1999 and 2012	91	20.9	21 (18-23)	7 (5-8)	18 (14-22)	12 (8-16)
			P = 0.002	P < 0.001	P < 0.001	P = 0.001
Diabetes						
Never	423	90.0	22 (19-24)	7 (6-8)	20 (16-24)	13 (11-17)
1999 and 2012	37	7.9	21 (17-23)	7 (5-8)	19 (14-22)	11 (9-14)
2012	10	2.1	19.5 (19-22)	7.5 (6-9)	17 (15.2-19.8)	10 (7-13.8)
			P = 0.112	P = 0.085	P = 0.183	P = 0.002

^*^P-value from Kruskal-Wallis rank sum test.

Further analysis using linear regression showed that there were no or minimal differences in cognitive performance between the two sexes. The scores from all tests showed small decreases with increasing age and clear increases with higher levels of education. For example, the scores in the learning test decreased by 1.70 words (95% confidence interval, CI: -2.15 to -1.25) for every 10 years of aging; and by 4.82 words (95% CI: -6.01 to -3.63) in a comparison between 14+ years and 8-10 years of schooling. The analyses on the coefficient of determination (R^2^) demonstrated that a higher level of education had a positive impact on cognitive test scores, such that they were raised by 8% to 14%. On the other hand, aging by 10 years had an inverse influence on cognitive test scores, such that they were lowered by 3% to 10% (Table 2).

**Table 2.** Associations (linear regression coefficients) between cognitive test scores and sociodemographic characteristics. Pró-Saúde study, Brazil, 2012

Cognitive tests	Coefficient (95% confidence interval)	R^2 a^	Coefficient (95% confidence interval)	R^2^
Learning				
Female^b^	0.94 (0.28 to 1.59)	0.01	1.05 (0.46 to 1.64)	0.20
Aging (10 years)	-1.70 (-2.15 to -1.25)	0.10	-1.17 (-1.62 to -0.71)	
11-14 years of schooling^c^	-1.95 (-2.6 to -1.29)	0.14	-1.6 (-2.25 to -0.95)	
8-10 years of schooling^c^	-4.82 (-6.01 to -3.63)		-3.8 (-5.03 to -2.58)	
Recall				
Female	0.28 (-0.06 to 0.61)	0.003	0.33 (0.02 to 0.64)	0.16
Aging (10 years)	-0.84 (-1.07 to -0.61)	0.09	-0.59 (-0.83 to -0.35)	
11-14 years of schooling	-0.8 (-1.14 to -0.46)	0.12	-0.62 (-0.96 to -0.98)	
8-10 years of schooling	-2.35 (-2.97 to -1.73)		-1.83 (-2.47 to -1.19)	
Semantic test (animals)				
Female	-1.32 (-2.33 to -0.31)	0.01	-1.16 (-2.10 to -0.22)	0.15
Aging (10 years)	-2.53 (-3.23 to -1.83)	0.09	-1.83 (-2.55 to -1.10)	
11-14 years of schooling	-3.05 (-4.08 to -2.01)	0.10	-2.46 (-3.49 to -1.43)	
8-10 years of schooling	-5.38 (-7.26 to -3.49)		-3.74 (-5.68 to -1.79)	
Phonemic test (letter F)				
Female	0.02 (-0.80 to 0.83)	-0.002	0.15 (-0.64 to 0.93)	0.08
Aging (10 years)	-1.16 (-1.74 to -0.58)	0.03	-0.64 (-1.24 to -0.03)	
11-14 years of schooling	-2.33 (-3.17 to -1.49)	0.08	-2.13 (-2.99 to -1.28)	
8-10 years of schooling	-3.6 (-5.13 to -2.07)		-3.04 (-4.66 to -1.42)	

^a^Adjusted R^2^; ^b^Male as a reference; ^c^14+ years of school as a reference.

CI = confidence interval.

The multiple logistic regression analysis showed that there was no statistically significant association between lowered cognitive performance and smoking or current alcohol use. After adjustments for sex, age and schooling, being physically active was found to be inversely associated with lowered cognitive performance in the recall memory test both in 1999 and in 2012 (odds ratio, OR = 0.29; 95% CI: 0.12-0.71). Both in 1999 and in 2012, reported diabetes was associated with worse performance in the phonemic verbal fluency test (OR = 5.81; 95% CI: 1.49-22.74), whereas among those with a first diagnosis in 2012, there was a borderline association with lower performance in the same test (OR = 1.88; 95% CI: 0.77-4.55). Both in 1999 and in 2012, reported hypertension was associated with lowered cognitive performance in the late memory test (recall, OR = 2.10; 95% CI: 1.07-4.13) and executive function test (phonemic test, OR = 3.16; 95% CI: 1.49-6.69). Among individuals with a diagnosis of hypertension in 2012, there was a significant association with lowered executive function (phonemic test, OR = 2.24; 95% CI: 1.01-4.98) (Table 3).

**Table 3.** Association between lowered cognitive performance and cardiovascular risk factors. Pró-Saúde study, Brazil, 2012

Variables	Learning	Recall	Semantic test	Phonemic test
	Odds ratio (95% confidence interval)*			
Physical activity				
Never	1.0	1.0	1.0	1.0
1999	0.14 (0.04-0.42)	0.46 (0.22-0.95)	0.57 (0.27-1.22)	0.27 (0.11-0.71)
2012	0.86 (0.41-1.83)	0.29 (0.12-0.71)	0.61 (0.28-1.35)	0.48 (0.21-1.12)
1999 and 2012	0.82 (0.40-1.68)	0.71 (0.36-1.40)	0.80 (0.39-1.65)	0.71 (0.33-1.53)
Hypertension				
Never	1.0	1.0	1.0	1.0
1999	2.2 (0.71-6.83)	3.03 (1.00-9.22)	0.89 (0.26-3.09)	2.19 (0.6-7.91)
2012	0.99 (0.45-2.19)	1.37 (0.65-2.86)	0.56 (0.24-1.33)	2.24 (1.01-4.98)
1999 and 2012	0.92 (0.44-1.95)	2.10 (1.07-4.13)	1.21 (0.62-2.37)	3.16 (1.49-6.69)
Diabetes				
Never	1.0	1.0	1.0	1.0
1999 and 2012	0.40 (0.05-3.36)	0.78 (0.15-3.97)	0.34 (0.04-2.81)	5.81 (1.49-22.74)
2012	2.01 (0.88-4.58)	1.83 (0.83-4.07)	1.58 (0.67-3.71)	1.88 (0.77-4.55)

*After adjustment for sex, age and schooling.

## DISCUSSION

Our findings highlighted associations between lowered cognitive performance and age, schooling, physical activity, diabetes and hypertension among middle-aged Brazilian adults.

Cognitive decline is the result of long-term pathological processes that probably start around 45 years of age. Mild cognitive impairment or dementia becomes more prevalent among elderly people.^20^ However, recent cohort studies have also shown cognitive dysfunctions among middle-aged adults.^3^^,^^21^ Because the populations of Brazil and other low and middle-income countries (LMIC) are becoming older, the epidemic of dementia is expected to expand into these regions, where it remains understudied.^22^

The observed beneficial effect of physical activity on cognition was expected, given that several previous studies have documented its beneficial influence on brain function.^21^ The small number of current smokers (10%) in this study may explain the absence of any association between smoking and cognitive performance.

Our study corroborates the association that was found between presence of diabetes and lower cognitive performance among the participants of the Brazilian Longitudinal Study of Adult Health (Estudo Longitudinal de Saúde do Adulto, ELSA-Brasil), which also included civil servants at educational institutions and used the same battery of cognitive tests.^6^ Moreover, in ELSA-Brasil, measurement invariance was analyzed via multiple-group confirmatory factor analysis: the findings suggested that differences in cognitive performance based on those tests indicated true differences.^23^

Lower cognitive performance is a plausible marker for chronic vascular diseases, given that diabetes and hypertension cause vascular damage and cognitive impairment. This association corroborates the vascular hypothesis of Alzheimer’s disease, first proposed in 1993. This suggested that cardiovascular risk factors are likely to play a critical role in cognitive decline during aging.^2^ Two recent reviews on the effects of diabetes and hypertension on cognitive performance have reinforced these associations.^24^^,^^25^ Cardiovascular and carotid artery diseases can cause chronic brain hypoperfusion many years before any symptoms of cognitive impairment are seen.^2^ Cognitive dysfunction in diabetes is associated with multiple disorders, such as chronic hyperglycemia, microvascular disease, altered sensorium, cortical atrophy and abnormalities in white matter tracts, along with abnormalities of brain metabolites.^20^

Other studies have also shown that education has greater influence on cognition than does age.^17^^,^^26^ Although some cognitive tests that are less dependent on education have been proposed in Brazil, the Pró-Saúde study used international standardized tests that had been validated for use in Brazil, in order to be able to compare our results with those from other cohort studies around the world. Further studies are important for enabling better investigation of associations between the risks of cardiovascular diseases and cognitive performance among less educated people.

The strengths of this study include (a) its choice of an adult or middle-aged population from a middle-income country; and (b) its methodological rigor in data collection. However, the study limitations include its small sample and its cross-sectional design, which prevented us from making solid causal inferences between cognitive performance and the associated variables. Importantly, our analyses did not consider the length of time since the medical diagnosis of diabetes and hypertension had been made. Nor did it consider disease severity or successful disease control. Moreover, this study did not include cognitive tests on attention and mental speed. Given that diabetes is associated with mental and motor slowing, less severe cognitive decline may also be associated with these cognitive functions.^21^

## CONCLUSIONS

Aging, low schooling level and cardiovascular risk factors may represent disadvantages over the course of life that operate through biological and social mechanisms and result in lowered cognitive performance and cognitive decline.^27^ Since currently there are no effective disease-modifying cures or treatments for the majority of the diseases that lead to dementia, prevention is crucial for decreasing the burden of dementia on patients and society.^1^ Prevention should start early and be continued throughout the individual’s life scan, as already recommended for other chronic diseases.
